# Multiple Bismuth Quadruple Therapy Containing Tetracyclines Combined with Other Antibiotics and *Helicobacter pylori* Eradication Therapy

**DOI:** 10.3390/jcm11237040

**Published:** 2022-11-28

**Authors:** Yingchao Sun, Mengjia Zhu, Lei Yue, Weiling Hu

**Affiliations:** 1 Department of Gastroenterology, Sir Run Run Shaw Hospital, Medical School, Zhejiang University, Hangzhou 310016, China; 2 Institute of Gastroenterology, Zhejiang University (IGZJU), Hangzhou 310027, China; 3 Zhejiang University Cancer Center, Hangzhou 310029, China

**Keywords:** Helicobacter infection, tetracycline, eradication, penicillin allergy, furazolidone, metronidazole, amoxicillin

## Abstract

*Helicobacter pylori* (HP) infection is closely associated with the development of chronic gastritis, peptic ulcer, and gastric cancer. However, the resistance rate of *H. pylori* strains to antibiotics such as clarithromycin, metronidazole, and levofloxacin has increased significantly, resulting in a significant decrease in the eradication efficacy of commonly used regimens. Tetracycline has received the attention of domestic and foreign scholars because of its low resistance. The purpose of this review is to provide an update on the tetracycline-containing bismuth quadruple eradication therapy for *H. pylori* infection and review the efficacy and safety of the regimens, hoping to provide guidance for clinical practice.

## 1. Introduction

*Helicobacter pylori* (HP) infection is closely associated with the development of chronic gastritis, peptic ulcers, dyspepsia, gastric mucosa-associated lymphoid tissue lymphoma, and gastric cancer, and approximately 75% of gastric cancers worldwide can be attributed to *Helicobacter pylori*-induced inflammation and injury [1]. China is a country with a dual high incidence of *Helicobacter pylori* infection and gastric cancer, with a current infection rate as high as 50%, and the number of cases of gastric cancer accounts for about 44% worldwide [2]. The Kyoto Global Consensus on HP Gastritis proposes that HP infection is an infectious disease, and all those who are positive for HP infection should receive eradication therapy [3]. However, the resistance rate of *H. pylori* strains to antibiotics such as clarithromycin, metronidazole, and levofloxacin has increased significantly, resulting in a significant decrease in the eradication efficacy of commonly used regimens [4].

Tetracycline was discovered in the 1940s and exhibited activity against a wide range of microorganisms, including *Helicobacter pylori*. It binds reversibly to a pocket in the 30S subunit of bacterial ribosomes containing 16S rRNA, causing bacteriostatic and bactericidal effects by inhibiting protein synthesis and bacterial growth [5]. Tetracycline has received the attention of domestic and foreign scholars because of its low resistance and high eradication efficacy; therefore, the Maastricht VI consensus recommended in 2022 that PPI-bismuth-tetracycline-metronidazole be used to eradicate HP during first-line treatment regardless of clarithromycin resistance [6]. According to Italy’s latest guidelines, bismuth-based quadruple therapy (BQT) should be used as first-line treatment for *H. pylori* in Italy for patients with high (>15%) or unknown prevalence of clarithromycin resistance [7]. The sixth expert consensus in China also recommends a bismuth quadruple regimen as the first and rescue therapy for HP-infected individuals, and the non-bismuth quadruple regimen (concomitant regimen, heterozygous regimen, and sequential regimen) is no longer recommended. Bismuth is a mucosal protective agent that can eradicate HP by inhibiting the adhesion of HP and inhibiting its protease, urokinase and phospholipase [8]. Bismuth has no drug resistance and has high safety in short-term applications. Recommended tetracycline-containing bismuth quadruple regimens include tetracycline (500 mg three times daily/four times daily) combined with metronidazole (400 mg three times daily/four times daily) or amoxicillin (1000 mg twice daily).

The purpose of this review is to provide a summary of the tetracycline-containing bismuth quadruple eradication therapy used to treat HP infection. In the last decade, the importance of the infection and the advantages associated with its eradication have become increasingly recognized [9,10]. Treatment regimens for HP infection with eradication rates greater than 90% are generally regarded as successful [11]. As resistance to other antibiotics increases, tetracycline has been increasingly investigated for its effectiveness in eradication therapy, but it also has a higher incidence of adverse effects. Therefore, this article also reviews the safety of tetracycline-containing bismuth quadruple regimens, hoping to provide guidance for clinical practice.

## 2. Eradication Rates, Safety, and Compliance with Bismuth-Containing Tetracycline Quadruple Therapy

### 2.1. Tetracycline + Metronidazole

Due to the low rate of HP resistance to tetracycline, several countries or regions in Europe and the United States recommend bismuth quadruple therapy, commonly known as classical quadruple therapy, including PPI-bismuth-tetracycline-metronidazole, as a first or rescue therapy for HP eradication [6]. It has been shown that *H. pylori* eradication rates were less than 80% with the 7-day regimen, 88.9% with the 10-day regimen for the intention-to-treat (ITT) analysis, and 91.6% for the per-protocol (PP) analysis [12,13]. In 2020, the Spanish team explored the effectiveness of metronidazole combined with tetracycline as a third-line treatment in patients with refractory infections who had failed both clarithromycin and levofloxacin, with eradication rates of 82% and 83% by ITT and PP analysis, respectively. These rates were decreased by 5–10% in patients who had previously used metronidazole [14]. In 2013, a single-center randomized controlled trial (RCT) in China using tetracycline combined with furazolidone or amoxicillin or metronidazole for 14 days achieved an eradication rate of more than 90% in rescue therapy patients with HP infection [15]. Among them, the ITT and PP analysis of tetracycline combined with metronidazole regimen were 87.9% and 93.1%, and tetracycline combined with furazolidone achieved higher eradication rates with ITT and PP values of 91.6% and 96.7%, respectively. However, tetracycline combined with furazolidone also had a higher incidence of adverse events in patients compared with the other two regimens (33.6%). A real-life study has been conducted in Italy where BQT provided eradication rates higher than 90%, even in areas with high clarithromycin resistance, with an ITT and PP of 91.5% and 95.8%, respectively [16]. A single-center retrospective study in the northeast region of Poland investigated the effectiveness of BQT 10-day therapy in the eradication of *H. pylori* infection, resulting in an eradication rate of 89.4% (84/94), which also provided a good efficacy [17].

Pylera is a 3-in-1 capsule containing 140 mg bismuth subcitrate, 125 mg metronidazole, and 125 mg tetracycline. In a meta-analysis of 21 studies, Pylera, as a first-line therapy, resulted in approximately 90% eradication in an ITT analysis [18]. The incidence of clarithromycin resistance is approximately 30% in some regions of central and southern Italy, and several studies conducted in Italy have confirmed the high efficacy of Pylera across a high prevalence of clarithromycin (CLA) resistance regions [19,20,21]. A prospective, uncontrolled, single-center observational study of 200 treatment-naïve HP-infected patients conducted by the Spanish team achieved high eradication rates with four times daily Pylera capsules combined with PPI twice in the morning and evening for 10 consecutive days, with rates of 91.5% and 95.2% in the ITT and PP analyses [22]. A real-life study conducted in Italy drew a similar conclusion that BQT using Pylera is an effective treatment strategy, with ITT eradication rates higher than 90%, even in areas with high resistance rates to *H. pylori* strains CLA or CLA + metronidazole resistance [23].

### 2.2. Tetracycline + Furazolidone

Furazolidone is a nitrofuran antibiotic with strong antioxidant activity and good absorption and distribution characteristics. In addition, antibiotic resistance is unlikely to develop. Other regimens containing furazolidone have previously been reported to achieve relatively high eradication rates [24,25]. Previous retrospective studies have shown that bismuth quadruple therapy containing tetracycline and furazolidone regimens could provide good eradication, with eradication rates of 91.74% and 95.24% in ITT and PP analyses, but retrospective studies have a low level of evidence [26]. In northwest China, a multicenter RCT study was conducted in 2020 to investigate the non-inferiority of amoxicillin plus berberine vs. tetracycline plus furazolidone quadruple therapy for HP rescue therapy, and the ITT and PP analyses were 77.5% and 85% [27]. Previous Chinese studies have also reported 7-day therapy with tetracycline (750 mg twice daily) combined with furazolidone (1000 mg twice daily), but good eradication rates have not been achieved, with ITT and PP only 68.6% and 72.7% [28]. Another retrospective study using Lactobacillus acidophilus (1 g three times daily) for 2 weeks followed by a bismuth-containing quadruple regimen (tetracycline 750 mg twice daily + furazolidone 100 mg twice daily) for 10 days as rescue therapy in patients who had previously eradicated *H. pylori* twice or more showed values of 92% and 91.8% in ITT and PP analyses, respectively, suggesting that patients with multiple eradications of infection may benefit from probiotic therapy [29].

### 2.3. Tetracycline + Amoxicillin

A meta-analysis explored the efficacy of tetracycline combined with amoxicillin for *H. pylori* eradication and included 33 studies, with ITT and PP analysis of 78.1% and 84.5%, and the relative risk (OR) of this regimen was 0.9 compared with other regimens, suggesting that tetracycline plus amoxicillin may not be inferior to other studies, but more clinical studies are needed to verify this [30]. In 2018, a randomized controlled trial (RCT) in China using rabeprazole (10 mg twice daily), bismuth, amoxicillin (1000 mg twice daily), and tetracycline (750 mg twice daily) 10-day quadruple therapy as first-line treatment for *H. pylori* eradication showed eradication rates of 87.2% and 91.9% in ITT and PP analyses, respectively, and efficacy was not affected by antibiotic resistance [31]. Although some studies have shown that amoxicillin combined with tetracycline is pharmacologically resistant, this clinical study still achieved a high eradication rate. Several studies previously also reported that the efficacy of tetracycline combined with an amoxicillin bismuth quadruple regimen is unsatisfactory. In a 2016 Korean study using pantoprazole 40 mg, bismuth 600 mg, tetracycline 1 g, and amoxicillin 1 g twice daily as first-line therapy for HP infection, the ITT and PP analyses were only 68.7% and 76.5%, and there was a 36.9% incidence of adverse events [32].

### 2.4. Tetracycline + Levofloxacin

In China, due to the many clinical applications of quinolones, there is cross-resistance with levofloxacin. At present, the resistance rate of levofloxacin in China has reached more than 20% [33]. Therefore, the guidelines do not recommend levofloxacin-containing regimens as first-line treatments unless applied to patients allergic to penicillin [34]. The Maastricht VI consensus indicated in 2022 that, regardless of clarithromycin resistance, eradication of HP with PPI-bismuth-tetracycline-levofloxacin is recommended when rescue therapy is given [6]. The Taiwan team has explored that a 10-day tetracycline (500 mg four times daily) regimen combined with levofloxacin (500 mg once daily) quadruple bismuth-containing regimen for HP-infected patients who failed sequential therapy provides a better eradication rate, achieving values of 95.8% in both ITT and PP analyses, but the sample size was small, with only 24 patients, and more studies are needed to confirm these findings [35]. In a 2019 RCT study (NCT02978157) conducted by the CHINA Taiwan team, 50 patients were treated for second-line HP infection with esomeprazole 40 mg twice daily, bismuth 120 mg once daily, tetracycline 500 mg once daily, and levofloxacin 500 mg every other day. There was a 22% rate of adverse events, primarily nausea and dizziness, but results regarding eradication rates were not published.

In Table 1, we summarize the efficacy and safety of tetracycline-containing bismuth quadruple regimens and list the specific study regimens, durations, and sample sizes. 

## 3. Tetracycline Regimen Selection for Penicillin-Allergic Patients

Amoxicillin is one of the most effective antibiotics against *Helicobacter pylori*, and the resistance rate is low, but about 10% of patients are allergic to penicillin, making it difficult to use these drugs in these patients. Maastricht VI consensus and the sixth Chinese consensus recommended that PPI-bismuth-tetracycline-metronidazole was first recommended to eradicate HP in patients allergic to penicillin during first-line treatment [6]. In 2018, a retrospective study in China using tetracycline (500 mg three times daily) combined with metronidazole (400 mg three times daily) as the first-line regimen included 120 patients with penicillin allergy and achieved a high eradication rate of 86.7% and 94.5% in ITT and PP analyses, respectively [37]. However, the incidence of adverse reactions was 44%, and 10 patients discontinued treatment due to adverse reactions. Although the total dosage of tetracycline and metronidazole in this study was lower than the dosage of antibiotics used in previous domestic studies, the efficacy was not disappointing [13]. In the past, two groups applied first-line 10-day regimens of PPI, tetracycline, and metronidazole in 5 and 17 patients with penicillin allergy, respectively, and reported eradication rates of 80–85%, but the number of patients was small, and more studies are needed to verify the reliability of this conclusion [38,39]. Quadruple therapy (PPI, bismuth, tetracycline, and metronidazole) is generally recommended as the best second-line therapy for HP infection after the failure of standard PPI-based triple therapy [40]. The Spanish team treated patients with penicillin allergy with tetracycline combined with metronidazole in a 10-day regimen for first-line treatment; 50 patients were included, but the eradication rate was less than 80% [41].

Although both the Maastricht consensus and the American Gastroenterological Association consensus recommend tetracycline combined with bismuth metronidazole quadruple regimen for rescue therapy, most retrospective studies have a low level of evidence and generally small sample size, and more studies are needed to verify its efficacy. In 2020, the Spanish team used data from the European *Helicobacter pylori* Administrative Registry to reassess the efficacy and safety of first and rescue therapy for HP-infected patients, and the classical bismuth quadruple regimen achieved a high eradication rate in first-line therapy in 250 patients, with values of 91% and 92% in the ITT and PP analyses. Classical bismuth quadruple therapy as second-line therapy also provided eradication rates of 78% and 82% in patients previously treated with triple therapy (PPI + clarithromycin + metronidazole). The eradication rate of this regimen was 77.8% at third-line treatment. Adverse events were higher with bismuth quadruple therapy in the first-line setting (29% of cases). Dysgeusia, diarrhea, and nausea were the most common adverse events, and the duration varied from 5 to nearly 10 days [42]. In Table 2, we summarize the efficacy and safety of tetracycline-containing bismuth quadruple therapy for penicillin-allergic patients and list the specific treatment lines, regimens, durations, and sample sizes.

## 4. Precautions for Using Tetracycline

Tetracycline is a broad-spectrum antibiotic produced by actinomycetes that inhibits bacterial reproduction and viability by interfering with protein synthesis. However, previous studies have shown that the incidence of individual adverse reactions is unsatisfied, and common adverse drug reactions to tetracycline include gastrointestinal symptoms (nausea, vomiting, and epigastric discomfort), photosensitivity reactions, allergy, anaphylactic shock, asthma, and hemolytic anemia. Loss of appetite, severe liver damage, and affected tooth and bone growth have been reported in studies. In previous studies, quadruple therapy with (PPI + bismuth + tetracycline + furazolidone) in penicillin-allergic patients developed more severe adverse reactions (drug fever, rash) in 5.5% of patients, although the eradication rate of the regimen (ITT 91.7%, PP 95.2%) exceeded 90% [26]. Tetracycline–metronidazole-containing quadruple regimens had a lower incidence of serious adverse reactions compared with tetracycline–furazolidone-containing quadruple regimens. Bismuth quadruple therapy with tetracycline 1000 mg twice daily combined with metronidazole 500 mg three times daily for seven days is as effective and safe as quadruple therapy with conventional doses (tetracycline 500 mg four times daily combined with metronidazole 500 mg three times daily) as rescue therapy [36]. Abdominal pain, bloating, and other discomforts were less frequent with twice-daily doses [36]. Common adverse events and the reported frequency of each regimen are listed in Table 3.

## 5. Recent Advances

Following the formal recognition of HP gastritis as an infectious disease in 2015, it was recommended that all patients should undertake eradication medication [3,9]. With infectious diseases, it is generally possible to reliably cure almost 100% of cases; however, the eradication rates reported in current studies are still far from this goal. In the future, *H. pylori* treatment trials will focus on actual cure rates, and comparisons will be restricted to deciding which of two highly successful therapies (average cure rate of at least 90%, preferably ≥95%) is best [11]. 

*H. pylori* therapies should be susceptibility based, relying either on susceptibility testing or on proven high local success rates. The prevalence of antibiotic resistance has increased such that clarithromycin, metronidazole, or fluoroquinolone triple therapies can no longer be used empirically. The first step in identifying and prescribing an effective therapy is to exclude antibiotics where preexisting resistance is likely. This can be accomplished by history and/or susceptibility testing, such as real-time PCR and even next-generation sequencing (NGS). Susceptibility-guided therapy (SGT) is an effective way to achieve high efficacy and avoid unnecessary antibiotic use while ensuring cost-effectiveness. Several clinical trials [44,45] were conducted to evaluate whether SGT shows a superior or similar efficacy in comparison with bismuth-containing quadruple therapy. It is necessary to carry out susceptibility-guided *H. pylori* eradication therapy versus empirical tetracycline-bismuth containing quadruple regimen in terms of eradication rate and cost in the future.

Vonoprazan (VPZ), a reversible H+-K+ ATPase inhibitor, has a fast and sustained acid suppression action that is unaffected by diet or polymorphisms in genes [46,47]. Vonoprazan increased the intragastric pH to over 4.0 within 4 h, which allowed the opportunity to achieve a shorter treatment duration [48]. VPZ-based regimens appear to be more effective than PPI-based regimens according to a present meta-analysis [49]. Vonoprazan is typically used in combination with amoxicillin or clarithromycin for *H. pylori* eradication therapy, and there have been no clinical trials involving its use in combination with tetracycline. VPZ’s excellent acid-suppressive ability combined with low tetracycline resistance will result in high eradication rates, but safety needs to be further evaluated in randomized controlled trials.

## 6. Conclusions

The HP infection rate is high, and drug resistance is prevalent worldwide. It is urgent to explore eradication regimens with high eradication rates, good safety, and good compliance. Tetracycline resistance is low in many countries, and tetracycline-containing bismuth quadruple regimens such as tetracycline combined with metronidazole or furazolidone or amoxicillin or levofloxacin can achieve better eradication rates. Especially in penicillin-allergic patients, tetracycline-containing bismuth quadruple regimens could be recommended as first-line therapy. However, current tetracycline-related studies were conducted mainly in Asia and Europe, and there are many retrospective studies. The efficacy and safety still need to be further evaluated by large-sample, multicenter prospective studies and more real-world data. Tetracycline has higher rates of adverse effects than other antibiotics, and current guidelines still recommend 1.5–2.0 g of tetracycline daily for eradication therapy. It is necessary to explore the effectiveness of lower doses of tetracycline-containing bismuth quadruple regimens in the future.

## Figures and Tables

**Table 1:** Studies of tetracycline-containing bismuth quadruple regimens in HP-infected patients.

Year	Author	Study	Regimen	Duration (d)	Sample Size	ER (ITT/PP, %)	AE (%)	Compliance (%)	Ref
2020	Nyssen et al.	Rescue, RCT	0 + T + M + B	14	45	82/83	63	96	[14]
2018	Xie et al.	1, RCT	R + T + A + B	14	109	87.2/91.9	5.5	97.2	[31]
2013	Lu et al.	Rescue, RCT	L + T + A + B	14	105	83.8/94.6	16.2	88.6	[15]
			L + T + M + B		107	87.9/93.1	33.6	94.4	
			L + T + F + B		108	91.7/96.1	17.6	95.4	
2020	Zhang et al.	Rescue, RCT	E + T + F + B	14	329	77.5/85	26.1	90	[27]
2014	Zhang et al.	Rescue, R	R + T + F + B	14	109	91.74/95.24	32.1	/	[26]
2014	Hsu et al.	Rescue, RCT	E + T + L + B	10	24	95.4/95.4	25	100	[35]
2016	Lee et al.	1, RCT	P + T + A + B	14	195	68.7/76.5	36.9	87.2	[32]
2020	Kim et al.	Rescue, R	P + T + M + B	7	98	92.9	36.7	/	[36]
2020	Fernandez et al.	1, RCT	PPI + Pylera^®^	10	200	91.5/95.2	28.5	96	[22]

R, rabeprazole; L, lansoprazole; E, esomeprazole; O, omeprazole; T, tetracycline; M, metronidazole; F, furazolidone; A, amoxicillin; L, levofloxacin; B, bismuth; ITT, intention-to-treat analysis; PP, per protocol analysis; RCT, randomized controlled trial; R, retrospective study; AE, adverse event; ER, eradication rate.

**Table 2:** Summary of studies with tetracycline-containing regimens in patients with penicillin allergy.

Year	Author	Treatment Line	Regimen	Duration (d)	Sample Size	ER (ITT/PP, %)	AE (%)	Ref
2020	Nyssen OP et al., RCT	1	PPI + T + M + B	14	250	91/92	29	[42]
		2		14	69	78.2/81.8	32	
		3		14	18	77.8/93.4	39	
2015	Gisbert et al., RCT	1	O + T + M + B	10	50	74/75	14	[41]
		2			24	37	58	
		3			3	100	67	
2018	Gao et al., R	1	R + T + A + B	14	120	86.7/94.5	47	[37]
2005	Rodrigue et al., R	1	E + T + M	10	17	82	/	[39]
		2			3	100	/	
2006	Matsushima et al., R	1	PPI + T + M	7–14	5	80	/	[38]
2005	Gisbert et al., RCT	2	RBC + T + M	7	17	47	53	[43]

R, rabeprazole; E, esomeprazole; O, omeprazole; PPI, proton pump inhibitors; RBC, ranitidine bismuth citrate; T, tetracycline; M, metronidazole; A, amoxicillin; B, bismuth; ITT, intention-to-treat analysis; PP, per protocol analysis; RCT, randomized controlled trial; R, retrospective study; AE, adverse event; ER, eradication rate.

**Table 3:** Adverse events of tetracycline-containing bismuth quadruple eradication therapy.

Adverse Effect	Reported Frequency			
	T + M [14,15]	T + A [15,31,32,37]	T + F [15,26,27]	T + L [35]
Taste disturbance	1.9–35%	5.6%	0–19.8%	4.2%
Nausea	15.9–41%	2.9–8.3%	2.8–33.7%	4.2%
Diarrhea	2.8–15%	0.46–16.7%	0.9–18.6%	4.2%
Reduced appetite	7.1–8.4%	1.4–1.9%	0–2.8%	4.2%
Vomiting	18%	2.8%	0–17.4%	4.2%
Abdominal pain	5.9–8.4%	1.9–9.2%	0–23.3%	4.2%
Headache/dizziness	3.7%	2.76–10%	1.9–9.3%	8.4%
Rash	0	1.9–3.3%	1.9–3.67%	4.2%
Fatigue	7.5–12%	1–5%	6.5–10.5%	4.2%
Fever	0	0.8–1.9%	1.9–5.5%	0
Bloating	0	15.3%	9.3%	0

T, tetracycline; M, metronidazole; F, furazolidone; A, amoxicillin; L, levofloxacin.

## Data Availability

Not applicable.

## References

[1] Amieva M., Peek R.M. (2016). Pathobiology of *Helicobacter pylori*-Induced Gastric Cancer. Gastroenterology.

[2] Gaskin T.A., Isobe J.H., Mathews J.L., Dillard D.R. (1988). The peel away introducer for the peritoneal limb of peritoneal venous shunt placement. Surg. Gynecol. Obstet..

[3] Sugano K., Tack J., Kuipers E.J., Graham D.Y., El-Omar E.M., Miura S., Haruma K., Asaka M., Uemura N., Malfertheiner P. (2015). Kyoto global consensus report on *Helicobacter pylori* gastritis. Gut.

[4] Undar A., Wang S. (2013). Translational research is a necessity for selecting the best components of the extracorporeal circuitry for neonatal and pediatric CPB patients. Perfusion.

[5] Chopra I., Roberts M. (2001). Tetracycline antibiotics: Mode of action, applications, molecular biology, and epidemiology of bacterial resistance. Microbiol. Mol. Biol. Rev..

[6] Malfertheiner P., Megraud F., Rokkas T., Gisbert J.P., Liou J.M., Schulz C., Gasbarrini A., Hunt R.H., Leja M., O’Morain C. (2022). Management of *Helicobacter pylori* infection: The Maastricht VI/Florence consensus report. Gut.

[7] Romano M., Gravina A.G., Eusebi L.H., Pellegrino R., Palladino G., Frazzoni L., Dajti E., Gasbarrini A., Di Mario F., Zagari R.M. (2022). Management of *Helicobacter pylori* infection: Guidelines of the Italian Society of Gastroenterology (SIGE) and the Italian Society of Digestive Endoscopy (SIED). Dig. Liver Dis..

[8] Lambert J.R., Midolo P. (1997). The actions of bismuth in the treatment of *Helicobacter pylori* infection. Aliment. Pharmacol. Ther..

[9] El-Serag H.B., Kao J.Y., Kanwal F., Gilger M., LoVecchio F., Moss S.F., Crowe S.E., Elfant A., Haas T., Hapke R.J. (2018). Houston Consensus Conference on Testing for *Helicobacter pylori* Infection in the United States. Clin. Gastroenterol. Hepatol..

[10] Liou J.M., Malfertheiner P., Lee Y.C., Sheu B.S., Sugano K., Cheng H.C., Yeoh K.G., Hsu P.I., Goh K.L., Mahachai V. (2020). Screening and eradication of *Helicobacter pylori* for gastric cancer prevention: The Taipei global consensus. Gut.

[11] Lee Y.C., Dore M.P., Graham D.Y. (2022). Diagnosis and Treatment of *Helicobacter pylori* Infection. Annu. Rev. Med..

[12] Lu H., Zhang W., Graham D.Y. (2013). Bismuth-containing quadruple therapy for *Helicobacter pylori*: Lessons from China. Eur. J. Gastroenterol. Hepatol..

[13] Zheng Q., Chen W.J., Lu H., Sun Q.J., Xiao S.D. (2010). Comparison of the efficacy of triple versus quadruple therapy on the eradication of *Helicobacter pylori* and antibiotic resistance. J. Dig. Dis..

[14] Nyssen O.P., Perez-Aisa A., Rodrigo L., Castro M., Mata Romero P., Ortuno J., Barrio J., Huguet J.M., Modollel I., Alcaide N. (2020). Bismuth quadruple regimen with tetracycline or doxycycline versus three-in-one single capsule as third-line rescue therapy for *Helicobacter pylori* infection: Spanish data of the European *Helicobacter pylori* Registry (Hp-EuReg). Helicobacter.

[15] Liang X., Xu X., Zheng Q., Zhang W., Sun Q., Liu W., Xiao S., Lu H. (2013). Efficacy of bismuth-containing quadruple therapies for clarithromycin-, metronidazole-, and fluoroquinolone-resistant *Helicobacter pylori* infections in a prospective study. Clin. Gastroenterol. Hepatol..

[16] Romano M., Gravina A.G., Nardone G., Federico A., Dallio M., Martorano M., Mucherino C., Romiti A., Avallone L., Granata L. (2020). Non-bismuth and bismuth quadruple therapies based on previous clarithromycin exposure are as effective and safe in an area of high clarithromycin resistance: A real-life study. Helicobacter.

[17] Wasielica-Berger J., Gugnacki P., Mlynarczyk M., Rogalski P., Swidnicka-Siergiejko A., Antonowicz S., Krzyzak M., Maslach D., Dabrowski A., Daniluk J. (2022). Comparative Effectiveness of Various Eradication Regimens for *Helicobacter pylori* Infection in the Northeastern Region of Poland. Int. J. Environ. Res. Public Health.

[18] Nyssen O.P., McNicholl A.G., Gisbert J.P. (2019). Meta-analysis of three-in-one single capsule bismuth-containing quadruple therapy for the eradication of *Helicobacter pylori*. Helicobacter.

[19] Zagari R.M., Romiti A., Ierardi E., Gravina A.G., Panarese A., Grande G., Savarino E., Maconi G., Stasi E., Eusebi L.H. (2018). The "three-in-one" formulation of bismuth quadruple therapy for *Helicobacter pylori* eradication with or without probiotics supplementation: Efficacy and safety in daily clinical practice. Helicobacter.

[20] Tursi A., Franceschi M., Allegretta L., Savarino E., De Bastiani R., Elisei W., Baldassarre G., Ferronato A., Scida S., Miraglia C. (2018). Effectiveness and Safety of Pylera(R) in Patients Infected by *Helicobacter pylori*: A Multicenter, Retrospective, Real Life Study. Dig. Dis..

[21] Di Ciaula A., Scaccianoce G., Venerito M., Zullo A., Bonfrate L., Rokkas T., Portincasa P. (2017). Eradication rates in Italian subjects heterogeneously managed for *Helicobacter pylori* infection. Time to abandon empiric treatments in Southern Europe. J. Gastrointest. Liver Dis..

[22] Castro Fernandez M., Romero Garcia T., Keco Huerga A., Pabon Jaen M., Lamas Rojas E., Llorca Fernandez R., Grande Santamaria L., Rojas Feria M. (2019). Compliance, adverse effects and effectiveness of first line bismuth-containing quadruple treatment (Pylera(R)) to eradicate *Helicobacter pylori* infection in 200 patients. Rev. Esp. Enferm. Dig..

[23] Gravina A.G., Priadko K., Granata L., Facchiano A., Scida G., Cerbone R., Ciamarra P., Romano M. (2021). Single Capsule Bismuth Quadruple Therapy for Eradication of *H. pylori* Infection: A Real-Life Study. Front. Pharmacol..

[24] Cheng H., Hu F.L. (2009). Furazolidone, amoxicillin, bismuth and rabeprazole quadruple rescue therapy for the eradication of *Helicobacter pylori*. World J. Gastroenterol..

[25] Eisig J.N., Silva F.M., Barbuti R.C., Rodriguez T.N., Malfertheiner P., Moraes Filho J.P., Zaterka S. (2009). Efficacy of a 7-day course of furazolidone, levofloxacin, and lansoprazole after failed *Helicobacter pylori* eradication. BMC Gastroenterol..

[26] Zhang Y., Gao W., Cheng H., Zhang X., Hu F. (2014). Tetracycline- and furazolidone-containing quadruple regimen as rescue treatment for *Helicobacter pylori* infection: A single center retrospective study. Helicobacter.

[27] Zhang J., Han C., Lu W.Q., Wang N., Wu S.R., Wang Y.X., Ma J.P., Wang J.H., Hao C., Yuan D.H. (2020). A randomized, multicenter and noninferiority study of amoxicillin plus berberine vs tetracycline plus furazolidone in quadruple therapy for *Helicobacter pylori* rescue treatment. J. Dig. Dis..

[28] Yang J.C., Lu C.W., Lin C.J. (2002). Rescue therapy for treatment failure of *Helicobacter pylori* infection. Chin. J. Gastroenterol..

[29] Liu A., Wang Y., Song Y., Du Y. (2020). Treatment with compound Lactobacillus acidophilus followed by a tetracycline- and furazolidone-containing quadruple regimen as a rescue therapy for *Helicobacter pylori* infection. Saudi J. Gastroenterol..

[30] Lv Z.F., Wang F.C., Zheng H.L., Wang B., Xie Y., Zhou X.J., Lv N.H. (2015). Meta-analysis: Is combination of tetracycline and amoxicillin suitable for *Helicobacter pylori* infection?. World J. Gastroenterol..

[31] Xie Y., Zhu Z., Wang J., Zhang L., Zhang Z., Lu H., Zeng Z., Chen S., Liu D., Lv N. (2018). Ten-Day Quadruple Therapy Comprising Low-Dose Rabeprazole, Bismuth, Amoxicillin, and Tetracycline Is an Effective and Safe First-Line Treatment for *Helicobacter pylori* Infection in a Population with High Antibiotic Resistance: A Prospective, Multicenter, Randomized, Parallel-Controlled Clinical Trial in China. Antimicrob. Agents Chemother..

[32] Lee J.Y., Kim N., Park K.S., Kim H.J., Park S.M., Baik G.H., Shim K.N., Oh J.H., Choi S.C., Kim S.E. (2016). Comparison of sequential therapy and amoxicillin/tetracycline containing bismuth quadruple therapy for the first-line eradication of *Helicobacter pylori*: A prospective, multi-center, randomized clinical trial. BMC Gastroenterol..

[33] Su P., Li Y., Li H., Zhang J., Lin L., Wang Q., Guo F., Ji Z., Mao J., Tang W. (2013). Antibiotic resistance of *Helicobacter pylori* isolated in the Southeast Coastal Region of China. Helicobacter.

[34] Liu W.Z., Xie Y., Lu H., Cheng H., Zeng Z.R., Zhou L.Y., Chen Y., Wang J.B., Du Y.Q., Lu N.H. (2018). Fifth Chinese National Consensus Report on the management of *Helicobacter pylori* infection. Helicobacter.

[35] Hsu P.I., Chen W.C., Tsay F.W., Shih C.A., Kao S.S., Wang H.M., Yu H.C., Lai K.H., Tseng H.H., Peng N.J. (2014). Ten-day Quadruple therapy comprising proton-pump inhibitor, bismuth, tetracycline, and levofloxacin achieves a high eradication rate for *Helicobacter pylori* infection after failure of sequential therapy. Helicobacter.

[36] Kim J.Y., Lee S.Y., Kim J.H., Sung I.K., Park H.S. (2020). Efficacy and safety of twice a day, bismuth-containing quadruple therapy using high-dose tetracycline and metronidazole for second-line *Helicobacter pylori* eradication. Helicobacter.

[37] Gao W., Zheng S.H., Cheng H., Wang C., Li Y.X., Xu Y., Hu F.L. (2019). Tetracycline and metronidazole based quadruple regimen as first line treatment for penicillin allergic patients with *Helicobacter pylori* infection. Zhonghua Yi Xue Za Zhi.

[38] Matsushima M., Suzuki T., Kurumada T., Watanabe S., Watanabe K., Kobayashi K., Deguchi R., Masui A., Takagi A., Shirai T. (2006). Tetracycline, metronidazole and amoxicillin-metronidazole combinations in proton pump inhibitor-based triple therapies are equally effective as alternative therapies against *Helicobacter pylori* infection. J. Gastroenterol. Hepatol..

[39] Rodriguez-Torres M., Salgado-Mercado R., Rios-Bedoya C.F., Aponte-Rivera E., Marxuach-Cuetara A.M., Rodriguez-Orengo J.F., Fernandez-Carbia A. (2005). High eradication rates of *Helicobacter pylori* infection with first- and second-line combination of esomeprazole, tetracycline, and metronidazole in patients allergic to penicillin. Dig. Dis. Sci..

[40] Gisbert J.P., Pajares J.M. (2002). Review article: *Helicobacter pylori* "rescue" regimen when proton pump inhibitor-based triple therapies fail. Aliment. Pharmacol. Ther..

[41] Gisbert J.P., Barrio J., Modolell I., Molina-Infante J., Aisa A.P., Castro-Fernandez M., Rodrigo L., Cosme A., Gisbert J.L., Fernandez-Bermejo M. (2015). *Helicobacter pylori* first-line and rescue treatments in the presence of penicillin allergy. Dig. Dis. Sci..

[42] Nyssen O.P., Perez-Aisa A., Tepes B., Rodrigo-Saez L., Romero P.M., Lucendo A., Castro-Fernandez M., Phull P., Barrio J., Bujanda L. (2020). *Helicobacter pylori* first-line and rescue treatments in patients allergic to penicillin: Experience from the European Registry on *H pylori* management (Hp-EuReg). Helicobacter.

[43] Gisbert J.P., Gisbert J.L., Marcos S., Olivares D., Pajares J.M. (2005). *Helicobacter pylori* first-line treatment and rescue options in patients allergic to penicillin. Aliment. Pharmacol. Ther..

[44] Zhou L., Zhang J., Song Z., He L., Li Y., Qian J., Bai P., Xue Y., Wang Y., Lin S. (2016). Tailored versus Triple plus Bismuth or Concomitant Therapy as Initial *Helicobacter pylori* Treatment: A Randomized Trial. Helicobacter.

[45] Chen Q., Long X., Ji Y., Liang X., Li D., Gao H., Xu B., Liu M., Chen Y., Sun Y. (2019). Randomised controlled trial: Susceptibility-guided therapy versus empiric bismuth quadruple therapy for first-line *Helicobacter pylori* treatment. Aliment. Pharmacol. Ther..

[46] Sue S., Maeda S. (2021). Is a Potassium-Competitive Acid Blocker Truly Superior to Proton Pump Inhibitors in Terms of *Helicobacter pylori* Eradication?. Gut Liver.

[47] Kiyotoki S., Nishikawa J., Sakaida I. (2020). Efficacy of Vonoprazan for *Helicobacter pylori* Eradication. Intern. Med..

[48] Jenkins H., Sakurai Y., Nishimura A., Okamoto H., Hibberd M., Jenkins R., Yoneyama T., Ashida K., Ogama Y., Warrington S. (2015). Randomised clinical trial: Safety, tolerability, pharmacokinetics and pharmacodynamics of repeated doses of TAK-438 (vonoprazan), a novel potassium-competitive acid blocker, in healthy male subjects. Aliment. Pharmacol. Ther..

[49] Jung Y.S., Kim E.H., Park C.H. (2017). Systematic review with meta-analysis: The efficacy of vonoprazan-based triple therapy on *Helicobacter pylori* eradication. Aliment. Pharmacol. Ther..

